# Waning of Maternal Antibodies against Measles Suggests a Large Window of Susceptibility in Infants in Lao People’s Democratic Republic

**DOI:** 10.3390/pathogens10101316

**Published:** 2021-10-13

**Authors:** Phonepaseuth Khampanisong, Maude Pauly, Phonethipsavanh Nouanthong, Molly A. Vickers, Siriphone Virachith, Kinnaly Xaydalasouk, Antony P. Black, Claude P. Muller, Judith M. Hübschen

**Affiliations:** 1 Institut Pasteur du Laos, Samsenthai Road, Ban Kao-Gnot, Sisattanak District, Vientiane P.O. Box 3560, Laos; kpnzpaul@gmail.com (P.K.); thip_mt@hotmail.com (P.N.); kawaii-nina@hotmail.com (S.V.); kinnaly_xaydalasouk@wvi.org (K.X.); a.black@pasteur.la (A.P.B.); 2 Department of Infection and Immunity, Luxembourg Institute of Health, 29, rue Henri Koch, 4354 Esch-sur-Alzette, Luxembourg; paulymaude@gmx.net (M.P.); molly.a.vickers@gmail.com (M.A.V.); claude.muller@lih.lu (C.P.M.)

**Keywords:** Lao PDR, maternal antibodies, measles, seropositivity, waning

## Abstract

Introduction: Measles is an endemic but largely neglected disease in Lao People’s Democratic Republic. New-borns are protected by maternal antibodies, but antibody waning before measles vaccination at 9 months of age leaves infants susceptible to infection. In this study, the susceptibility window of infants was determined to generate scientific evidence to assess the national measles immunization strategy. Methods: Between 2015 and 2016, demographic data, medical history, and blood samples were collected from 508 mother-child pairs at the provincial hospital in Vientiane. The samples were screened with a commercial kit detecting anti-measles IgG antibodies. Results: The large majority (95.7%) of the mothers were seropositive for anti-measles IgG and antibody titers of the mothers and infants were highly correlated (*p* < 0.01). While at birth 97.7% of the infants were seropositive, seropositivity rates decreased to 74.2% two months later to reach only 28.2% four months after birth (*p* < 0.01). Just before the first dose of the measles-rubella vaccine, scheduled at 9 months of age, was actually given, less than 14% of the infants were seropositive. Conclusion: This alarmingly wide susceptibility gap due to rapid maternal antibody decay leaves infants at risk of measles infection and serious disease complications. A high herd immunity is crucial to protect young infants and can be achieved through improved routine vaccination coverage and (expanded age group) supplementary immunization activities.

## 1. Introduction

In developing countries, measles remains one of the leading causes of death among infants under five years of age. Since the advent of the vaccine in the sixties, the burden of measles has decreased substantially worldwide [1]. The first dose of measles-containing vaccine (MCV1) is administered at 9 or 12 months, depending on the countries’ transmission levels and risks of measles mortality in infants [2]. The second dose, MCV2, should be given between 15 and 18 months to ensure both a high seroconversion rate and long-term protection [3]. Owing the high transmissibility of measles virus, a vaccination coverage of at least 95% is considered necessary to achieve population immunity [4].

Infants of measles-seropositive mothers are protected by maternal antibodies that were transmitted transplacentally to the foetus. Gestational age of the child, maternal nutrition, as well as maternal vaccination and infection status determine the level of antibodies passively acquired by the child and as a result the duration of post-natal protection [5,6]. Early waning of antibodies results in an extended window of susceptibility that lasts until the child reaches the age of recommended vaccination [7]. If the vaccine is given too early, immaturity of the immune system as well as neutralizing maternal antibodies prevent successful seroconversion [3,8]. Nevertheless, in special circumstances including outbreaks and supplementary immunization activities (SIAs), measles vaccination can be administered to infants from 6 months [3]. Vaccination before 9 months is considered a supplementary dose (recorded as MCV0) and therefore both MCV1 and MCV2 are still recommended. While there have been suggestions to permanently lower the age for delivery of MCV1 to 6 months in low-income countries to decrease the risk of measles outbreaks [9,10,11], more research is needed to assess benefits versus drawbacks. Such a programme shift would probably incur additional costs and logistical efforts, adding to the burden on low-income countries [12]. 

In Lao People’s Democratic Republic (Lao PDR), the Expanded Program on Immunization (EPI) was launched in the early eighties. MCV1 is scheduled at 9 months and MCV2 at 12 to 18 months of age [13]. In 2018, WHO and UNICEF estimated national MCV1 coverage as 69% and MCV2 coverage as 57% [14]. A strategic plan developed by the Lao Ministry of Health outlines the steps to achieve 95% coverage for both doses by 2022 [15]. Although five nationwide measles vaccination campaigns were conducted since 2000, a high regional disparity in immunization coverage subsists [16,17] and the effectiveness of some SIAs was likely inadequate [16]. From 2016 to 2018, a total of 21 measles cases (8, 3, and 10 respectively) were reported, while a resurgence in 2019 resulted in 1037 confirmed and clinically compatible cases of measles, many of which were in children under five [15,18]. Since measles surveillance and reporting remain suboptimal in Lao PDR, with probably many undetected cases and those suspected not commonly confirmed in the laboratory, these figures may underestimate the real measles burden in the country [19,20,21].

In this study, measles IgG antibody presence and titers were assessed in Lao infants at birth and at different routine vaccination time points to determine their window of susceptibility to measles infection. 

## 2. Materials and Methods

### 2.1. Participants

This cross-sectional study was performed in the Vientiane Provincial Hospital, “Maria Teresa”, located in a semi-urban area, some 80 km north of Vientiane Capital. The hospital records 100–150 deliveries per month and approximately 80–100 infants are vaccinated per month as part of the national immunization program. 

Between May 2015 and December 2016, cord blood (CB) samples were obtained from 130 infants born in the hospital. In addition, blood was collected by heel-prick on filter paper (Dried Blood Spots-DBS) from 378 infants without history of measles infection, who attended the paediatric unit for routine immunizations with the pentavalent (diphtheria, pertussis, tetanus, and hepatitis B and Haemophilus influenzae type b, DPT-HepB-Hib) vaccine recommended at weeks 6, 10, and 14 or MCV1 vaccination recommended at 9 months of age. Blood of the mothers was collected on the same day as for their infants. There was no longitudinal follow-up and all tests were carried out on specimens from different individuals.

Personal data was collected by standardized questionnaire covering mother-related (i.e., date of birth, recall of previous measles infection/fever with rash episode and of vaccination history, educational level, body mass index (BMI), ethnicity, and profession) and child-related information (i.e., date of birth, sex, birth weight, and breastfeeding status of the infants).

All mothers gave written informed consent (or oral consent, in the case of illiterate women) and the study was approved by the Lao National Ethics Committee (reference numbers 029/2015/NECHR, 009/2016/NECHR, 077/2016/NECHR).

### 2.2. Serology 

After collection, maternal venous blood and cord blood was allowed to clot and serum was separated by centrifugation (2000× g for 10 min). All specimens were stored at 4 °C and transferred to Institut Pasteur du Laos within one week, where the samples were stored at −80 °C until further processing.

Elution from DBS samples was adapted from a previously described protocol [22]. In brief, 10 discs of 3 mm diameter (equivalent to approximately 12.5 µL of serum) were punched from the DBS and incubated in 250 µL of extraction buffer (PBS/0.5% Tween/5% dried skimmed milk powder) for 15 min at 50 °C. Following overnight incubation at 4 °C, samples were shaken for 15 min and centrifuged; 50 µL of the supernatant were used in the ELISA. 

All samples were screened with the Enzygnost Anti-Measles Virus/IgG ELISA (SIEMENS, Marburg, Germany) according to the manufacturer’s protocol. A cut-off value of 0.1 was used to differentiate corrected delta optical density (c∆OD) of positive/equivocal participants (hereafter “positive”) from negative participants. In addition to these qualitative results, antibody titers were only calculated for positive participants (c∆OD ≥ 0.1) according to the instructions provided by the kit manufacturer. c∆OD values obtained for DBS samples were multiplied by an adjustment factor (AF) generated as described before [23] to improve the comparability of the results of DBS and sera. The amount of DBS sample per participant was insufficient to allow retesting of participants with equivocal results.

### 2.3. Statistical Analysis

Descriptive and inferential statistics were performed in R studio using the *R Stats* package [24]. Bivariate analyses (i.e., Pearson’s Chi-Squared Test) were applied to assess which child- or mother-related sociodemographic factors (e.g., age, sex, weight, level of education, medical and vaccination history) affect the odds of seropositivity, as well as antibody levels. The *epitab* function of *epitools* [25] was used to calculate 95% confidence intervals (CI) and odds ratios (OR) of the chi-square statistics. The independent *t*-test, *t.test*() function, was used to test the statistical differences between the antibody titer means of the different vaccination time points. Linear regression was performed using the *lm*() function to assess whether maternal and infant antibody titers were correlated and whether infant antibody titers decreased with time. The *anova*() function from the *car* package [26] was used to compare regression models and report if they were significantly different. The figures were created with the *ggplot2* package [27]. 

## 3. Results

### 3.1. Evaluation of the DBS Elution Protocol

Paired serum and DBS samples of eight anti-measles IgG positive volunteers were tested in triplicates and in parallel on two different ELISA plates. The c∆OD of each volunteer was calculated as the mean c∆OD of the triplicates for the two plates.

The c∆OD obtained for DBS and paired serum samples were highly correlated (Adj R2 = 0.82, Intercept = 0.44, Slope = 1.36). The adjustment factor (AF) was generated as described before [23]:

$$AF=\sqrt{\frac{a{\;}_{\frac{c\Delta {\mathrm{OD}}_{\mathrm{serum}}}{c\Delta {\mathrm{OD}}_{\mathrm{DBS}}}}}{a_{\frac{c\Delta {\mathrm{OD}}_{\mathrm{DBS}}}{c\Delta {\mathrm{OD}}_{\mathrm{serum}}}}}}=1.94,$$

where *a* is the regression coefficient. The paired *t*-test showed that the mean differences between the c∆OD of serum and adjusted c∆OD of DBS were not significantly different. When dividing the c∆OD obtained for the sera by the c∆OD obtained for paired DBS samples, a similar adjustment factor of 2.13 was obtained. All c∆OD obtained from DBS in this study were multiplied by the adjustment factor 1.94.

### 3.2. Statistical Description of the Mother-Infant Pairs 

The majority of the mothers were of Lao Loum ethnicity (*n* = 472, 92.9%) and many had graduated from secondary school (*n* = 237, 46.7%). The mean age of the mothers was 27.5 years (age range: 16–48). The mean age of the infants depended on the sampling time point (DPT-HepB-Hib1: 7.5 weeks, range: 2–16; DPT-HepB-Hib2: 12.2 weeks, range: 7–22; DPT-HepB-Hib3: 18.0 weeks, range: 11–51; and MCV1: 41.3 weeks, range: 24–55). Thus, the majority of the infants were vaccinated later than recommended by the national immunization program. Although overall the delay for MCV1 vaccination was less dramatic than for the DPT-HepB-Hib vaccine, 61.7% infants were older than 10 months. Overall, 70.3% of the mothers remembered having received MCV, but only few remembered having had an episode of fever with rash (26.4%) or a clinically diagnosed measles infection (18.1%). Less than half of the mothers were primiparous (40.9%). The mean birth weight of the infants was 3.1 kg (95% CI 2.9–3.4). The sex ratio among the infants was equilibrated (female: 256 and male: 252).

### 3.3. Serology of the Mothers

Of the mothers, 95.7% were seropositive for anti-measles IgG with a median titer of 4024.2 mIU/mL (95% CI: 1609.0–8157.0). Although maternal age did not significantly influence the odds of anti-measles IgG seropositivity, antibody titers of the mothers slightly increased with age (*p* < 0.01; Table 1). Thus, primiparous mothers who were significantly younger than multiparous mothers also had somewhat lower titers (Table 1). Median titers in women who recalled an episode of fever with rash or a clinically diagnosed measles infection (hereafter “presumably naturally immune women”) were not significantly higher than in women who did not (Table 1). Presumably vaccinated (i.e., women who recalled having received MCV) and presumably naturally immune mothers were also not significantly more likely to be seropositive than the others (Table 1). None of the other recorded mother-related demographic variables (i.e., BMI, ethnicity, profession, and educational level) significantly influenced seropositivity rates or antibody titers of the mothers (data not shown). 

### 3.4. Serology of the Infants

**Figure 1:**
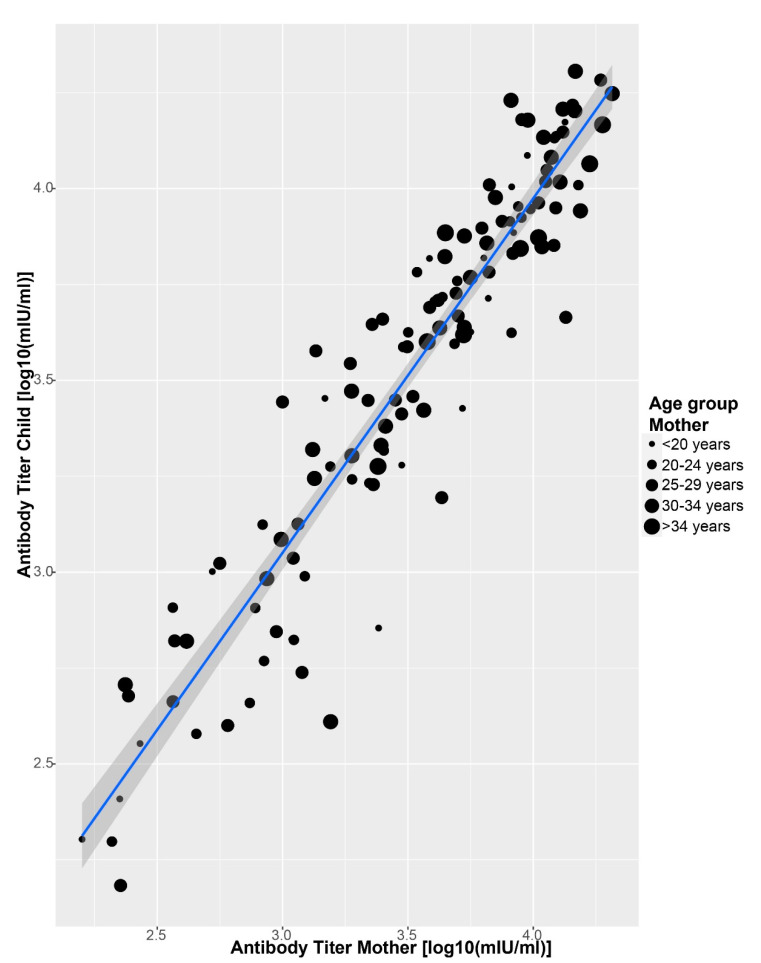
Correlation between log-transformed antibody titers of mothers and infants at birth and the effect of age of the mother (years). The blue line is the linear regression curve and the confidence interval is shaded (Adj R2 = 0.88, Intercept = 0.15, Slope = 0.96, *p* < 0.001).

Overall, 58.1% of the infants were seropositive for anti-measles IgG with adjusted median antibody titers of 773.7 mIU/mL (95% CI = 268.3–3819.2; Table 1). At birth, the IgG titers of the infants were highly correlated with those of the mothers (Adj R2 = 0.88, Intercept = 0.15, Slope = 0.96, *p* < 0.001; Figure 1). At the subsequent sampling time points, the correlation decreased. As anticipated, a significant positive correlation between age group of the mother and antibody titers of the infants was also revealed (*p* < 0.05; Figure 1). 

**Figure 2:**
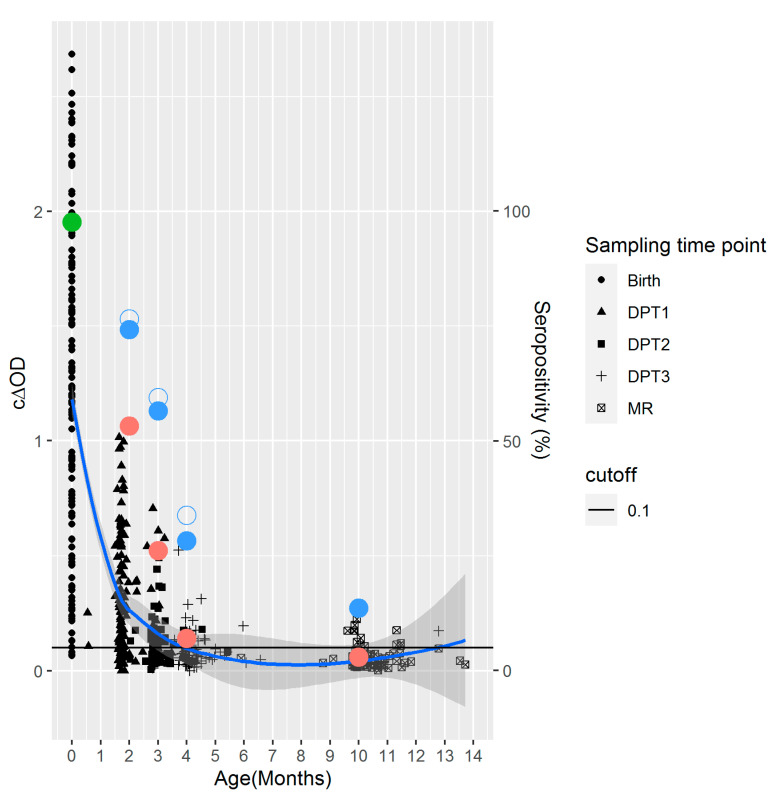
Waning of maternal antibodies after birth. Scatterplot of the corrected delta optical density (c∆OD) and the age (months) of the child. The different symbols represent the different sampling time-points. The colored large dots show the decreasing seropositivity rates at various time points: the green dot being the seropositivity rate at birth (in cord blood samples), the red dots being the seropositivity rates at months 2, 3, 4, and 10 (in dried blood spot samples-DBS), and the full and empty blue dots being the seropositivity rates at months 2, 3, 4, and 10 (in DBS) when applying an adjustment factor of 1.94 and 2.13, respectively. The blue line is the LOESS curve. Shaded are the confidence intervals. The horizontal black line is the cut-off value for c∆OD used in the ELISA to differentiate seropositives/equivocals from seronegatives for anti-measles antibodies (0.1).

At birth, the median IgG titer of the infants was 4332.1 mIU/mL (95% CI: 1698.8–8309.1) and 97.7% of the infants were seropositive. The three seronegative children were from seronegative mothers. At the scheduled vaccination time-points (DPT-HepB-Hib1-3 and MCV1), the titers decreased to 591.3, 246.2, 243.9, and 218.0 mIU/mL, respectively (Adj R2 = 0.12, Intercept = 2.81, Slope = 0.01, *p* < 0.001; Figure 2). In line with the decrease of antibody titers, the seropositivity rates decreased significantly with time and were as low as 13.8% at the MCV1 sampling time point (*p* < 0.01; Figure 2; Table 1). Since DPT-HepB-Hib1-3 and MCV1 were given during a wide infant age range, data were also analyzed according to months after birth. At 2 months, 74.2% (92/124) were seropositive for anti-measles IgG, while only 28.2% (20/71) were positive at 4 months (Figure 2). The rate of decline of the median c∆OD between months 2 and 4 was approximately 50% per month (Figure 2). In the following 6 months, the decay clearly slowed down: at month 4 the median c∆OD reached 0.07 and at month 10 it reached 0.04 (Figure 2).

## 4. Discussion

We assessed the presence of measles IgG antibodies in Lao mothers and infants by screening samples of mother-child pairs at different time points after birth. The Maria Teresa hospital was chosen because of the high number of patients it receives and the quality of care. We opted for DBS sampling at the vaccination time points as it is a suitable alternative specimen type for surveillance in countries such as Lao PDR, where refrigerated storage and transport of samples is challenging [28,29]. Our preliminary tests confirmed that DBS sampling is a viable alternative to serum sampling for anti-measles IgG surveillance when adjusting the c∆OD value. Our adjustment factor was similar to the one reported in a previous study using the same approach (i.e., 1.28 versus 1.94 in this study) [23]. 

Overall, over 95% of the women and new-borns were seropositive for measles IgG. As expected, antibody titers of the new-borns were positively and significantly correlated with those of their mothers. In contrast to some previous studies [30,31], median titers of presumably vaccinated women were similar to the titers of presumably naturally immune women. However, this finding needs very careful consideration as we relied on the memory of the mothers and had no access to supporting material (e.g., vaccination cards or laboratory reports). Interestingly, older mothers had higher seropositivity rates and antibody levels. Additionally, antibody titers of the infants were positively correlated with the age of the mother. This can possibly be explained by the higher number of mothers with natural immunity in the older age brackets. Individuals with past wild-type infection are thought to have significantly higher measles IgG antibody levels than those with vaccine-induced immunity and this is mirrored in their infant’s antibody concentration [3,30,32,33]. Moreover, several expanded age group SIAs (e.g., the 2011 SIA had a target age of 9 months to 19 years) were conducted in the past and may have included some of our participants, potentially leading to a boost in antibody concentrations [3]. 

Here, we showed that a high percentage of the infants were no longer protected before receiving their first measles vaccination. In fact, a rapid decrease in overall seropositivity rates was observed between the vaccination time points. Already four months after birth, more than 71% of the infants were negative for measles IgG antibodies and at the time of first measles vaccination (MCV1), more than 86% were negative. Similar findings were reported in measles elimination settings where the duration of protection by maternal antibodies lasted less than 4 months [30,34,35,36]. This is not a major problem when herd immunity rates are high, but in countries like Lao PDR the measles virus continues to circulate and surveillance is incomplete. The identified large window of susceptibility puts infants at risk, especially during high incidence years such as 2019, with 1037 confirmed cases [18]. An earlier administration of the first vaccine dose would shorten the susceptibility period. However, humoral immunogenicity is reduced when measles vaccination is administered at 6 months, with an average seroconversion rate of 76% compared with 92% at 9 months [37]. As a result, vaccine effectiveness has been demonstrated to be lower in those under 9 months but whether or not administration would still be beneficial in terms of reducing disease severity and overall morbidity is an ongoing discussion [2,11,37,38,39,40,41]. Meta-analysis suggests that antibody levels are reduced in infants immunized before 9 months even after subsequent MCV1 and MCV2 vaccination, while differences in seropositivity, T cell responses, and vaccine effectiveness are not seen [42]. The clinical and epidemiological impact of mild immune blunting in the form of antibody titers is unknown [42]. Furthermore, the mechanisms of maternal antibody interference are still debated and no specific maternal antibody titer that prevents seroconversion in the infant has been defined [43,44]. Thus, further high-quality research needs to be conducted to better tailor measles vaccination schedules in countries still struggling with high transmission and measles-associated mortality rates. 

We used a cut-off value of 0.1 to differentiate positive and negative participants. Although previous studies have used the same cut-off value, some have only considered samples with OD values of more than 0.2 as positive [23,30]. Additionally, once the AF was applied, the number of positives increased. Thus, we may even have overestimated the number of positives, suggesting that the real situation may be much worse than presented. Finally, while we replicated the techniques published for DBS sampling [23], results were very much dependent on the AF obtained and residual confounding may still be present. 

In conclusion, our study reveals an alarmingly wide susceptibility gap between maternal antibody decay and immunization for a high proportion of Lao infants. Based on current evidence, ensuring sufficient coverage, also via SIAs, is essential to protect these susceptible infants through herd immunity rather than advocating earlier vaccine administration. 

## Figures and Tables

**Table 1:** Mother- and child-related demographic characteristics and anti-measles IgG seropositivity.

Mother-Related Variables			Anti-Measles IgG Seropositivity		IgG Titer (mIU/mL)	
			*n*/N (%)	*p*-Value ^a^	Median (95% CI)	*p*-Value ^b^
**All time points**	Age groups (years)	<20	28/32 (87.5)	0.10	2695.8 (998.9–6418.0)	<0.01
		20–24	107/112 (95.5)		3346.6 (1233.4–7203.2)	
		25–29	184/188 (97.9)		3790.8 (1467.5–7988.0)	
		30–45	119/126 (94.4)		5302.0 (2533.0–10207.0)	
		>45	48/50 (96.0)		4587.8 (2298.2–7215.2)	
	Measles-containing vaccination recalled	No	141/151 (93.4)	0.15	4174.9 (1880.4-8603.2)	0.89
		Yes	345/357 (96.6)		3937.6 (1499.6–8135.7)	
	Fever rash recalled	No	339/357 (95.0)	0.22	4030.4 (1717.0–8279.6)	0.50
		Yes	130/134 (97.0)		5332.4 (1444.3–7431.1)	
		unknown	17		n.a.	
	Measles infection recalled	No	395/416 (95.0)	0.15	3878.9 (1563.2–8306.0)	0.79
		Yes	91/92 (98.9)		4166.0 (1754.0–7230.0)	
	Parity number	1	201/208 (96.6)	0.51	3721.0 (1353.9–7657.9)	0.09
		>1	285/300 (95.0)		4197.0 (1760.3–8652.7)	
	**Total**		486/508 (95.7)	n.a.	4024.2 (1609.0–8157.0)	n.a.
**Child-Related Variables**			**Anti-measles IgG Seropositivity**		**IgG Titer (mIU/mL)**	
			***n*/N (%)**	***p*** **-Value**	**Median (95% CI)**	***p*** **-Value**
**At birth (cord blood)**	Birth weight of child (g)	≤3000	53/54 (98.1)	1.00	4326.9 (1631.9–9682.2)	0.61
		>3000	74/76 (97.4)		3715.4 (1218.6–7314.6)	
	**Total**		127/130 (97.7)	n.a.	4332.1 (1698.8–8309.1)	n.a.
**Other time points (DBS ^c^**)	Vaccination schedule	DPT-HepB-Hib 1	102/139 (73.4)	<0.01	591.3 (295.8–1275.3)	<0.01
		DPT-HepB-Hib 2	36/63 (57.1)		246.2 (200.3–361.6)	
		DPT-HepB-Hib 3	20/82 (24.4)		243.9 (208.0–326.9)	
		MCV1	13/94 (13.8)		218.0 (170.2–276.6)	
	Breast feeding	No	14/51 (27.5)	0.16	360.9 (207.0–647.6)	0.35
		Yes	157/327 (48.0)		358.1 (214.2–831.0)	
	**Total**		171/378 (45.2)	n.a.	358.1 (213.7–808.1)	n.a.
**All time points**	**Total**		298/508 (58.7)	n.a.	807.9 (287.7–3369.3)	n.a.

*n* is the number of anti-measles IgG seropositives, N the total number of participants; n.a. not applicable; ^a^ assessed by Pearson’s Chi-squared test or Fisher’s Exact Test; ^b^ Welch Two Sample t-test or one-way ANOVA; ^c^ an adjustment factor of 1.94 was applied to the optical density values of dried blood spots (DBS).

## Data Availability

Due to ethical restrictions and participant confidentiality, the data set cannot be made publicly available.
